# pH Dependence of MRI Contrast in Magnetic Nanoparticle Suspensions Demonstrates Inner-Sphere Relaxivity Contributions and Reveals the Mechanism of Dissolution

**DOI:** 10.1021/acs.langmuir.2c02621

**Published:** 2023-02-03

**Authors:** Eoghan MacMahon, Dermot F. Brougham

**Affiliations:** School of Chemistry, University College Dublin, Belfield, Dublin 4, Ireland

## Abstract

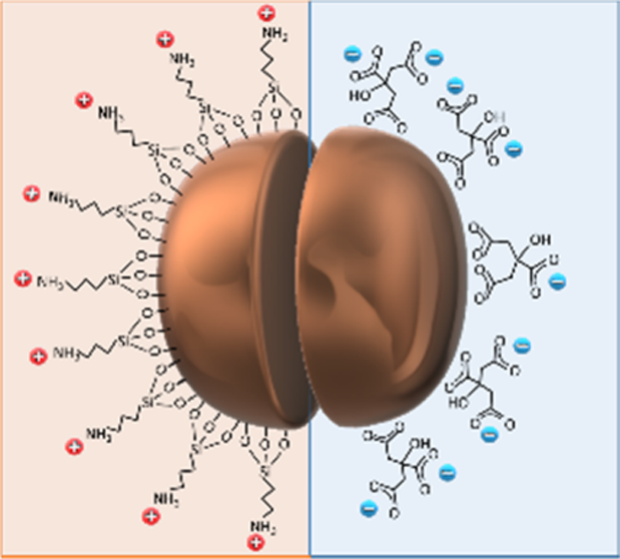

Superparamagnetic iron oxide nanoparticles, MNPs, are under investigation as stimulus-responsive nanocarriers that can be tracked by magnetic resonance imaging. However, fundamental questions remain, including the effect of differing surface chemistries on MR image contrast efficacy (relaxivity), both initially and over time in the biological environment. The effects of pH and ligand type on the relaxivity of electrostatically and sterically stabilized spherical 8.8 nm superparamagnetic MNP suspensions are described. It is shown for the first time that across the pH ranges, within which the particles are fully dispersed, increasing acidity progressively reduces relaxivity for all ligand types. This effect is stronger for electrostatically (citrate or APTES) than for sterically stabilized (PEG5000) MNPs. NMR relaxation profiles (relaxivity as a function of ^1^H Larmor frequency) identified an inner-sphere effect, arising from the protonation of bare oxide or low-molecular-weight-bound species, as the cause. The suppression is not accounted for by the accepted model (SPM theory) and is contrary to previous reports of increased relaxivity at lower pH for paramagnetic iron oxide nanoparticles. We propose that the suppression arises from the orientation of water molecules, with the oxygen atom facing the surface increasingly preferred with increasing surface protonation. For APTES-stabilized MNPs, pendant amines and the silane layer confer exceptional chemical and colloidal stability at low pH. Dissolution of these particles at pH 1.8 was monitored over several months by combining in situ measurements of relaxation profiles with dynamic light scattering. It was shown that particles are magnetically intact for extended periods until they rapidly dissolve, once the silane layer is breached, in a process that is apparently second order in particle concentration. The findings are of interest for tracking MNP fate, for quantitation, and for retention of magnetic responsiveness in biological settings.

## Introduction

Magnetic resonance imaging (MRI) is a key clinical technique due to its ability to distinguish between healthy and pathological tissue. Images are recorded of slices within which the contrast can reflect variation in local ^1^H density, but is primarily determined by the longitudinal (*T*_1_) and transverse (*T*_2_ or *T*_2_*) NMR relaxation times. The coupling of ^1^H spins with larger moments of electrons can significantly reduce the relaxation times. Hence, extensive efforts have been ongoing over several decades to develop contrast agents based on metal ions that have unpaired electrons, with differing pharmacological profiles, contrast generation ability, and responsiveness.

Clinically gadolinium(III)-based agents are used in *T*_1_-weighted MRI (providing local intensity enhancement).^1^ Clusters of dextran- and carboxydextran-stabilized superparamagnetic iron oxide nanoparticles (MNPs) were developed for *T*_2_-weighted imaging (local intensity suppression).^2,3^ These agents are usually rapidly opsonized and transported to the liver where they generate detectable contrast^4^ that has potential for staging liver disease.^5^ Several MNP-based agents were discontinued due to safety issues, so currently ferumoxytol (polyglucose sorbitol carboxymethylether-coated MNPs), which was developed as an imaging agent, is the only iron oxide product that has FDA approval, for anemia treatment in patients with chronic kidney disease.^6^ However, it is used off-label for MRI angiography in patients with renal failure.

Research in the field remains strong; for instance, Ferumoxytol offers possibilities for both *T*_1_- and *T*_2_-weighting modalities, and so it continues to be under evaluation as a versatile vascular contrast agent.^7,8^ Suspensions of fully dispersed citrate-stabilized MNPs (*d*_core_ 4 nm) have also been considered for *T*_1_-weighted angiography.^9,10^ MNPs of many types have been developed for stem cell tracking following uptake.^11−13^ There are also many exciting recent examples of advanced multiresponsive magnetic iron oxide particles. For instance, smaller particles (in the 2–3 nm range, so the cores have no appreciable moment and are paramagnetic rather than superparamagnetic) are being developed for dual *T*_1_–*T*_2_ contrast generation for evaluating vascular permeability in tumors.^14^ The same authors describe the assembly of MNPs (in the same size range, and identified as γ-Fe_2_O_3_) into colloidally stable constructs that disassemble at lower pH to generate local contrast that is detectable within tumor models under *T*_1_-weighting.^15^ MNPs are also a strong focus for developing next-generation theranostic agents because (i) they can have a better toxicity profile than the Gd(III) chelates;^16^ (ii) they respond strongly to external magnetic fields (magnetophoretic motion in DC-field gradients^17^ and hyperthermic heating in AC-fields^18^) providing multifunctionality; and (iii) their amenable surface chemistry can be used both to influence biodistribution and uptake and even to provide information on the local environment.^19,20^

Hence, the development of MNP-based agents for use in man is the subject of ongoing study and discussion.^21^ However, paramagnetic and superparamagnetic iron-oxide-based MRI agents have yet to reach their clinical potential. This is in part because of a lack of understanding of the effect of the biological environment combined with the surface chemistry on the water–MNP interactions that determine contrast, both initially and over time. For instance, in the case of cell therapies, the possibility of estimating MNP content in tissue following cellular uptake and transport to the lysosomes where the local pH is low requires knowledge of the effect of the environment on the relaxation.

The contrast generation efficacy of an agent is quantified by the relaxivities, *r*_1_ and *r*_2_, which are the ^1^H relaxation rate enhancements of the suspending medium per millimolar concentration of metal (the relaxation rates, *R*_1,2_, are the reciprocals of the times, *T*_1,2_). Relaxation arises due to time-modulated interactions between the ^1^H pool and the magnetic centers.^22^ The coupling can be dipolar or scalar, and the modulation arises from different dynamic processes. Relaxation contributions are usually categorized as outer sphere (OS) when the contact time with the agent is the water translational diffusional correlation time, and inner sphere (IS) when there is extended contact, *i.e.*, exchange processes. For Gd(III) agents, IS contributions often dominate, while for superparamagnetic MNPs, OS contributions are thought to be most important.

NMR relaxation profiles are commonly used for studying MNP suspensions. These are measurements of *r*_1_ as a function of ^1^H Larmor frequency, ν_L_, and hence field strength, *B*_0_ (since ν_L_ = γ_H_*B*_0_/2π, where γ_H_ is the ^1^H gyromagnetic ratio 2.675 × 10^8^ rad T^–1^ s^–1^). The profiles are very sensitive to changes in nanoparticle form and dispersion, hence they have become established for evaluating the magnetic/colloidal properties of intact suspensions.^23−25^ The *r*_1_ values are determined by the relative ^1^H-MNP moment dynamic modulation. At high frequency, this is driven by the Brownian process and the relevant timescale is the Brownian correlation time, τ_B_, for H_2_O diffusion past the MNPs. Hence, this part of the profile is sensitive to particle size. The low-frequency part is governed by the Néel dynamics of the MNP moments. The Néel correlation time, τ_N_, is determined by the magnetocrystalline anisotropy energy, Δ*E*_anis_, arising from intrinsic factors, *i.e.*, size, shape, phase, and crystallinity,^22,23^ and it may also have contributions from particle aggregation.^24−26^ The commonly applied SPM model,^22^ developed by the Muller group at Mons, reproduces the experimental relaxation profiles of most superparamagnetic agents very well using an OS-only approach.

There have been a few studies into MRI contrast for pH-responsive particulate agents. The Mons group reported increased *r*_1_, at all ν_L_, on reducing pH for paramagnetic hydrated iron oxide (ferric oxyhydroxide) nanoparticle suspensions of ferritin (a Fe storage protein, composed of an 8 nm ferrihydrite, 5Fe_2_O_3_-9H_2_O, core) and fercayl (dextran-coated akageneite, β-FeOOH, particles).^27,28^ In these cases, the relaxivity is weak, as the particles are paramagnetic, and so there is minimal OS contribution. It was suggested that surface protonation-enhanced proton exchange with bulk water, an IS effect, caused the enhancement. The Mons group also studied tetramethylammonium hydroxide-coated superparamagnetic MNPs (γ-Fe_2_O_3_, d_core_ 4.9 nm) at an unadjusted basic pH and after stabilization with citrate at neutral pH. The *r*_1_ of the latter (at 60 MHz) was suppressed. However, this was ascribed to partial aggregation, as higher *r*_2_ and hydrodynamic size, *d*_hyd_, were reported on citrate stabilization,^29^ as opposed to pH-dependent differences in surface ionization. Despite this extensive work and the relevance of effects that alter relaxivity in the development of new agents, the effect of pH on relaxivity for superparamagnetic MNP suspensions is not fully understood. Studies on systems, with differing surface chemistries and full particle dispersion across the pH range, are needed to provide physical insights into surface-mediated effects on relaxivity.^30^

In this work, aqueous superparamagnetic MNP suspensions were prepared surface-stabilized with citrate, APTES, and PEG to provide particles that around neutral pH bear negative (called here MNP@cit), positive (MNP@APTES), and near-zero (MNP@PEG) surface charge. The pH ranges within which the suspensions are fully dispersed and hydrolytically stable were determined, and fast-field-cycling NMR relaxometry was used to record the relaxation profiles across these ranges. For all three MNP types, *r*_1_ decreased at all ν_L_ on decreasing pH, contrary to the previous reports for paramagnetic particles.^27,28^ These responses are shown to arise from an IS interaction associated with the preferred molecular orientation of water molecules on encountering the protonated MNP surface. This effect is beyond the accepted SPM model for ^1^H relaxation due to superparamagnetic MNPs.^22^ Finally, the MNP@APTES suspensions were found to be very stable in lysosome-like conditions. Hence, MNP@APTES dissolution at pH 1.8 was evaluated over 4.6 months by recording the development of the relaxation profiles, and the likely dissolution mechanism was identified. The implications of both the pH dependence of relaxivity and MNP dissolution for quantitation and tracking of particle fate following uptake and for developing pH-sensitive probes are discussed.

## Results and Discussion

### Formation of Electrostatically Stabilized MNP Suspensions

**Figure 1:**
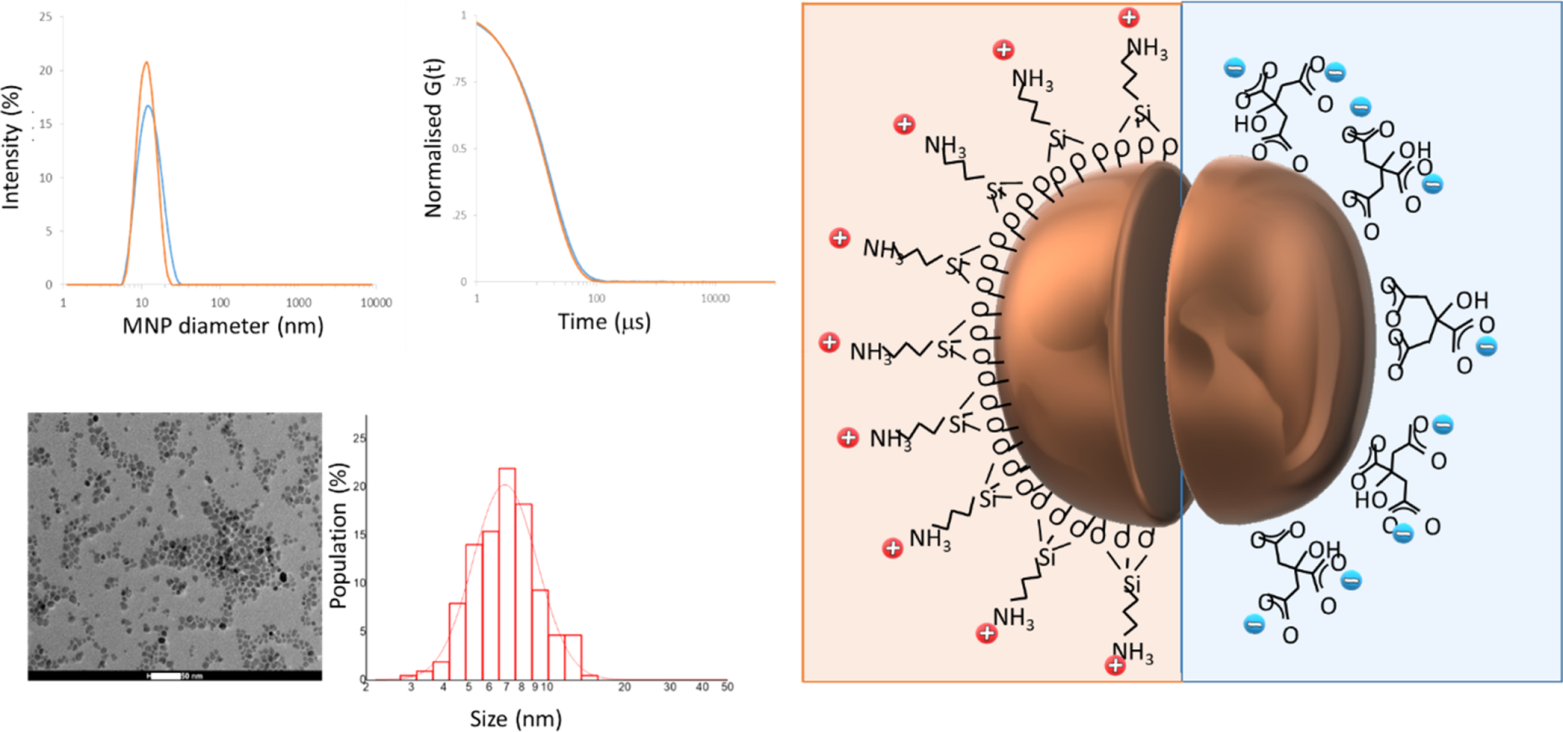
Left top, DLS number size distributions and correlograms for MNP@cit (blue) and MNP@APTES (red) suspensions, recorded at 25 °C. Left bottom, TEM analysis of MNP@OA, a log-normal fit gives *d*_TEM_ 9 ± 2 nm (*n* = 5409), scale bar 50 nm. Right, representation of the surface charges for aqueous MNP suspensions, emphasizing the identical MNP cores used in this study.

To provide MNP suspensions that differ only in the surface ligands, and so isolate the effects of surface chemistry on relaxivity, the surfactant-free thermal decomposition of iron acetylacetonate^31^ was selected. This established route provides easily functionalizable spherical MNPs of good crystallinity with excellent *T*_1_-contrast generation potential.^32^ Benzyl alcohol (BA) acts as a solvent and as a labile surfactant that can be replaced with multiple stabilizers.^33^ Stable suspensions in heptane of MNP@BA and MNP@OA (using oleic acid), and in water of MNP@cit, MNP@APTES and MNP@PEG (using sodium citrate tribasic, APTES, and PEG5000) were prepared, as described in the Methods section, using MNPs from batches with identical size and magnetic properties. The resulting organic and aqueous suspensions were colloidally stable. MNP@OA were used only for transmission electron microscopy, TEM, analysis, Figure 1, confirming the presence of spherical particles of size, *d*_TEM_ 9 ± 2 nm. We have previously established^34^ by X-ray absorption spectroscopy (XAS) that the oxide phase formed most closely resembles maghemite, γ-Fe_2_O_3_.

Dynamic light scattering (DLS) analysis of typical aqueous MNP@cit suspensions showed *Z*-average hydrodynamic size, *d*_hyd_, of 12 nm with an acceptable polydispersity index, *PDI*, of 0.20 at pH 7 (see Figure 1). While MNP@APTES gave *d*_hyd_ 11 nm, *PDI* 0.18 at pH 3.5. These two suspensions are almost indistinguishable by DLS with overlapping correlograms and no long-time features associated with aggregates. This strongly suggests full dispersion in both cases, which is confirmed by NMR, see below. Under these conditions the suspensions are quite stable, the *d*_hyd_ and *PDI* values do not change over weeks.

**Figure 2:**
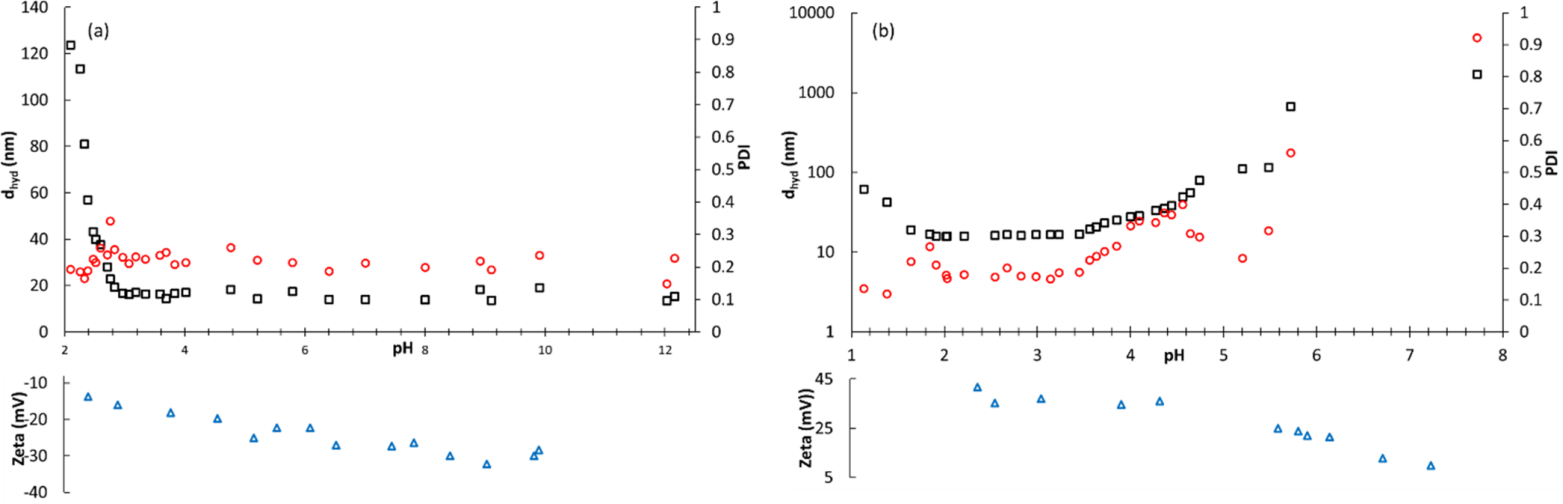
Colloidal characterization at 25 °C by dynamic light scattering and zeta potential (ζ) measurements (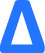) of typical aqueous electrostatically stabilized MNP suspensions. (a) MNP@cit, (b) MNP@APTES. The *Z*_ave_ (□) and *PDI* (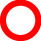) values are taken as a measure of *d*_hyd_ and the size polydispersity.

The pH of the MNP suspensions was adjusted using small aliquots of NaOH and HCl (both at 0.1 M) enabling the stable pH range to be determined, Figure 2. The *d*_hyd_ and *PDI* values of MNP@cit suspensions are low and unchanging at all pH above 2.8, and in this range, the zeta potential, ζ, value was strongly negative. ζ decreased gradually and quite smoothly, from −35 mV at pH 9.8 to −16 mV at pH 2.2, due to protonation of the pendant carboxylates and any free oxide, as has been previously described^29^ and consistent with theory.^35^ Features at pH values corresponding to the p*K*_a_ of free citrate (3.1, 4.8, and 6.4) are not apparent, possibly because citrate can bind to MNPs in a range of orientations. The colloidal stability observed for MNP@cit at neutral pH is a significant improvement on other reports. As noted above, for 4.9 nm MNP cores the Mons group observed a significant increase in *d*_hyd_ at neutral pH, from 8 to 18 nm,^29^ on stabilizing tetramethylammonium hydroxide-coated MNPs with citrate. For MNP@cit at pH lower than 2.8, the ζ value becomes less negative than −20 mV; the repulsive electrostatic interactions are weaker and the particles aggregate. Slow core dissolution also occurs in this pH range, although the suspensions are sufficiently stable for colloidal and NMR characterization, *i.e.*, the *d*_hyd_ and *r*_1_ values were unchanging on a given day.

For MNP@APTES suspensions, the *d*_hyd_ and *PDI* values are stable for extended times across a narrower pH range of 2.3–3.3, over which the ζ decreases from +42 to +35 mV. At higher pH, uncontrolled aggregation (increasing *d*_hyd_ and *PDI*) and rapid precipitation are observed presumably due to deprotonation of the pendant ammonium groups of bound APTES reducing interparticle electrostatic repulsion. At very low pH, MNP@APTES also gradually dissolve, although this is far slower than for MNP@cit. Dissolution of MNP@APTES at pH 1.8 is evaluated in detail in the Dissolution of MNP@APTES in suspension monitored by FFC-NMR section.

### pH-Dependent Magnetic Resonance Contrast

#### MNP@cit Suspensions

**Figure 3:**
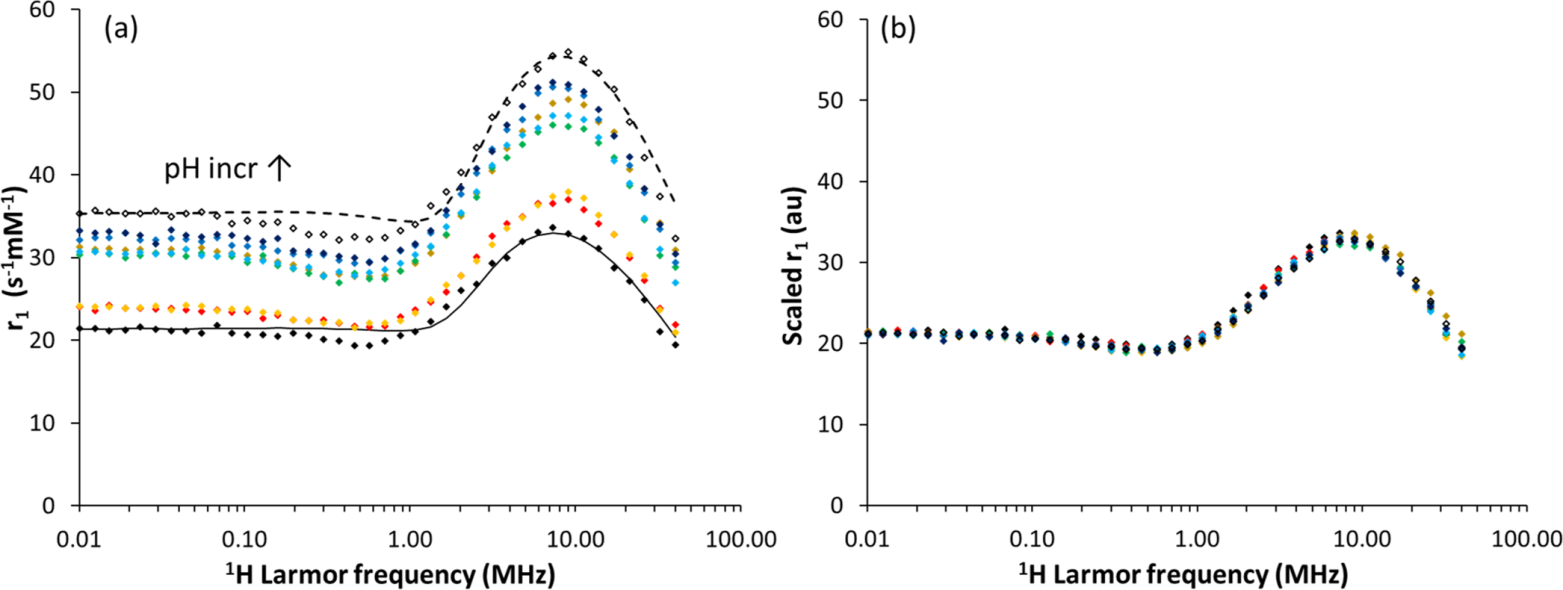
(a) pH-dependent FFC-NMR profiles of aqueous MNP@cit suspensions recorded at 25 °C as a function of pH (with increasing *r*_1_; pH 3.7, 5.3, 6.3, 6.8, 7.6, 9.3, 10.4, 12.0, 12.2). Simulated profiles are included as solid lines for pH 3.7 and 12.2, see text. (b) Same profiles scaled to a common *r*_1_ of 20 au at 0.01 MHz. The errors are of comparable size to the data markers, see the Methods section.

The pH dependence of the MRI relaxometric properties was assessed for MNP@cit suspensions across their wide pH-stable range. The spin-lattice relaxivities, *r*_1_, were measured as a function of ^1^H Larmor frequency using fast-field-cycling NMR relaxometry.^36^ FFC-NMR profiles of MNP@cit for a stock suspension of 5 mM Fe at (unadjusted) pH 6.4 and in the pH range between 3.7 and 12.2, are overlayed in Figure 3. The profiles show the characteristic shape, with a mid-frequency maximum, and low *r*_1_ at a low frequency that together confirm the superparamagnetic nature of MNP@cit and full particle dispersion.^22^ The *r*_1_ values are observed to decrease by *ca.* 13 s^–1^ mM^–1^ with decreasing pH over the range studied, *i.e.*, by *ca.* 37%. It should be emphasized that the *d_hyd_* and *PDI* values are unchanged in this pH range, Figure 1, and the changes in *r*_1_ do not arise due to dilution, which is taken into account in the determination of relaxivity.

Scaling all of the relaxation profiles to a common value, Figure 3, demonstrates that in this pH range, there is no change in shape. Hence, the rates of the dynamic processes (τ_N_ and τ_B_) that give rise to the spectrally dispersive features in the profiles are not altered by pH for fully dispersed MNP@cit. The ionic strength of the pH-adjusted suspensions is necessarily higher than that of the original suspension. Independent pH neutral suspensions were prepared, within the ionic strength range of those shown in Figure 3, by adding NaCl up to 1.13 mM. The profiles recorded at different ionic strengths are almost superimposable, Figure S1, and the hydrodynamic sizes were unchanged, confirming that the effects shown in Figure 3 are not due to changes in ionic strength. It is clear that the decrease in *r*_1_ with decreasing pH is due to increased protonation of the particle surface, *i.e.*, it is an IS effect.

SPM theory^22^ simulations of the FFC-NMR profiles for the highest and lowest pH suspensions are shown in Figure 3. These seem to provide adequate agreement with experimental values. The parameters obtained were, for pH 3.7: d_NMR_ 10.1 nm, M_S-NMR_ 55 emu g^–1^, τ_N_ 18 ns, Δ*E*_anis_ 1.8 GHz, and for pH 12.2: d_NMR_ 9.0 nm, M_S-NMR_ 77 emu g^–1^, τ_N_ 27 ns, Δ*E*_anis_ 1.7 GHz. The particle sizes, *d*_NMR_, and saturation magnetization, *M*_S-NMR_, values estimated from the simulations are broadly in the expected range, confirming again the superparamagnetic nature of the suspensions. However, with decreasing pH, the *M*_S-NMR_ value decreases from 77 to 55 emu g^–1^ and the *d*_NMR_ value increases from 9.0 to 10.1 nm, *i.e., it* is only possible to reproduce the experiment by altering the particle size. The picture suggested by the parameters obtained in this way is physically unrealistic, increased magnetic size should increase the magnetization, and as a result, the profile shape should change significantly. In addition, the simulations suggest a change in τ_N_, but there is no change in the profile shape. These issues underline a significant limitation of SPM theory; it does not account for IS effects, which in some cases, as shown here, can be a significant contribution to *r*_1_.

It is clear that the additional pH-dependent IS contribution is frequency-independent, *i.e.*, it contributes a constant *r*_1_ offset, in this ^1^H Larmor frequency window, and this increases with increasing pH. It is interesting that for MNP@cit, *r*_1_ does not change very smoothly with pH, *e.g*., the increase between pH 6.3 and 7.6 is notably strong, Figure 3a. This range coincides with the p*K*_a_ at 6.4 for one of the free citrate ionizations, confirming an *r*_1_ contribution from bound citrate and suggesting that relaxivity may be more sensitive than ζ to these effects. The presence of IS *r*_1_ contributions from protonation of bare oxide is also possible.

#### MNP@APTES Suspensions

**Figure 4:**
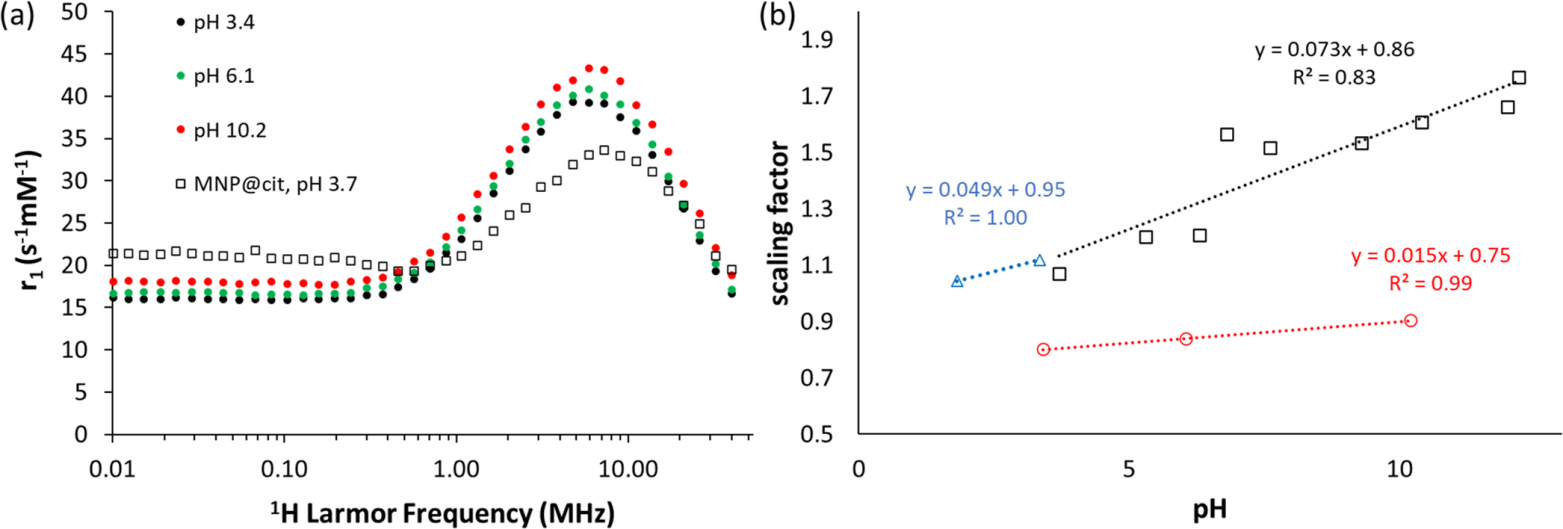
(a) FFC-NMR profiles recorded at 25 °C for MNP@PEG from pH 3.4 to 10.2; the pH 3.7 MNP@cit profile is also included. (b) Profile scaling factors (to 20 au at 0.01 MHz in all cases) as a function of pH for MNP@cit (□), MNP@APTES (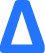), and MNP@PEG (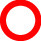); the scaled profiles are shown in Figures S2 and S3.

Profiles recorded for MNP@APTES suspensions at pH 1.8 and 3.4, *i.e.*, within the narrow stable pH window, show a weak decrease in *r*_1_, of 2–4 s^–1^ mM^–1^, with decreasing pH, Figure S2. Given that the p*K*_a_ of free APTES is *ca.* 10.8, the amine groups must be fully protonated across the range studied. So the pH dependence of *r*_1_ for MNP@APTES can be ascribed to the protonation of a fraction of free oxide. Scaling confirmed that the profile shape is again almost independent of pH, Figure S2. The accessible pH range in this case is narrow, but the pH dependencies, Figure 4b, allow us to tentatively suggest that the suppression is in the same range but somewhat weaker than for MNP@cit.

These observations suggest that the acidity-induced suppression for MNP@cit arises from the protonation of both bound citrate (*r*_1_ change at pH *ca.* 6.4) and bare oxide. It is clear that for both types of electrostatically stabilized MNPs, the *r*_1_ suppression arises from increased surface proton content, in whatever form.

#### MNP@PEG Suspensions

To prepare MNP@PEG, cores were grafted with 5 kDa silanized polyethylene glycol (PEG), see the Methods section. DLS analysis, Figure S3, shows unchanging *d*_hyd_ of 45 nm (*PDI* 0.19) at pH 3.4 and 10.2, a wider stable range, as expected. The *d*_hyd_ is larger than that of MNP@cit, which we attribute to the PEG chains. The FFC-NMR profiles were measured at pH 6.1 (unadjusted), 3.4, and 10.2, and the data are shown in Figure 4.

The profiles retain similar shape to those of the two electrostatically stabilized suspensions, and again a low *r*_1_ at low frequency confirms full particle dispersion.^22^ There is some broadening of the maximum, which we associate with fluctuating PEG chains. The relaxivity is very similar to that of MNP@cit (profile included in Figure 4) and indeed MNP@APTES (Figure S2), despite the greater *d*_hyd_ and presence of the chains. This confirms that the increased *d*_hyd_ is not due to any aggregation and shows that the key interactions with H_2_O that determine *r*_1_ for MNPs occur very close to the particle surface. For MNP@PEG, *r*_1_ was again found to decrease with decreasing pH (by *ca.* 15% between pH 10.2 and 3.3) and scaling confirmed that the profile shapes are again pH-independent, Figure S3, and similar to those for the electrostatically stabilized suspensions. Interestingly, the pH dependence of *r*_1_ is weaker, with slope (albeit inferred from only two data points) *ca.* 1/5th of that found for MNP@cit over a similar pH range, Figure 4. SPM simulations can reproduce the response, demonstrating the superparamagnetic nature of the particles but, as before, the pH dependence cannot be explained.

Given the methoxy end group used, the weak decrease in *r*_1_ with pH for MNP@PEG is likely to arise entirely from the protonation of a fraction of bare oxide. In a recent high-resolution ^1^H NMR study into PEG graft dynamics of SiO_2_ NPs in D_2_O,^37^ we showed that: (i) the achievable coverage with the ligand used in the current study, MeO-PEG5000-Si(OMe)_3_, is low at <1.0 chains nm^–2^, and (ii) at all PEG coverage sufficient to fully disperse the particles, while the chain ends are in the brush confirmation, a fraction of each chain (monomer units closer to the chain grafting point) collapse onto the particle surface. High-resolution ^1^H spectra cannot be obtained for MNPs. However, we suggest that for MNP@PEG, the surface presentation is similar with collapsed chain segments partially blocking water access to the oxide surface, and this effect also broadens the relaxation profile.

#### Effect of MNP Surface Protonation on Relaxivity

In summary, for the fully dispersed MNP suspensions with different surface chemistries studied, frequency-independent acidity-induced suppression of *r*_1_ is observed to some extent. This IS effect is due to the surface protonation of free oxide and bound ionizable groups (when present), which must be close to the surface. The suppression could be: (a) “dynamic” in origin, and/or it might be static, *i.e.*, arising from weaker ^1^H-moment coupling, due to (b) reduced particle magnetization and/or (c) increased distance of closest approach for ^1^H in H_2_O. Any very fast exchange process (a) would have to be faster than τ_B_, as the frequency of the MHz range *r*_1_ inflection, which is effectively τ_B_^–1^, is pH-independent. It is hard to imagine such a process as it would have to have a correlation time τ_c_ < 25 ns (or rate, τ_c_^–1^ > 40 × 10^6^ s^–1^) since anything slower would alter the high-frequency profile shape. *M*_s_ reduction (b) could arise from cores with a thicker outer nonmagnetic (disordered) oxide layer, due to stronger ligand binding on protonation. However, in that case, the profiles would also change shape with pH, so this possibility can also be excluded. Hence, the most likely explanation is the “static” view (c) that progressively increasing surface protonation progressively increases the average contact distance to the exchanging ^1^H of the H_2_O which, while not bound, are not OS.

**Scheme 1:**
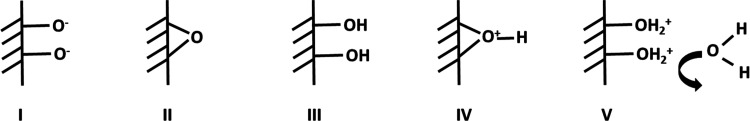
Candidate Surface Oxide Species of Increasing Prevalence (I → V) with Progressively Reducing pH For pristine, Fe_3_O_4_ p*K*_a_ values are reported^38^ as 9.0 (Fe=O^–^ ⇌ Fe=OH, *i.e.*, (I) ⇌ (III)) and as 4.4 (Fe=OH ⇌ Fe=OH_2_^+^, *i.e.*, (III) ⇌ (V)). Species V (Fe=OH_2_^+^) is suggested to be prevalent at low pH. Bridging oxygens (II, IV) may also play a role. The preferred water orientation at low pH is shown for species V.

For γ-Fe_2_O_3_, the fact that MNP@cit and MNP@APTES, with negative and positive ζ potentials, respectively, show similar strong acidity-induced *r*_1_ suppression demonstrates that the effect arises from close interaction of uncharged H_2_O, not H^+^, with the surface. As noted in the Introduction section, for suspensions of ferritin and fercayl (hydrated paramagnetic iron oxide nanoparticles), *r*_1_ increased weakly on reducing pH, by *ca.* 0.02 s^–1^ mM^–1^, over 4 pH units (a much lower, negative slope of −0.005, c/f Figure 4).^27,28^ Interestingly, the *r*_1_ enhancement was also observed at all ν_L_ and was attributed to increased H^+^ exchange on surface protonation through a Grotthus-like mechanism. For the paramagnetic phases, such an interaction can be easily envisaged on the relatively hydrogen-atom-rich oxyhydroxide surface, which will have surface hydration similar to that shown for species V, Scheme 1. This scheme shows the surface species anticipated for superparamagnetic iron oxide. In our case, the predominant species at intermediate pH may be bridging oxygens, species II.^38^ On decreasing pH, the surface population will shift toward species III, IV, and V. However, irrespective of the exact speciation at any given pH, as the surface ^1^H content increases, dipolar considerations will prefer the orientation of incoming water molecules with the oxygen pointed toward the surface, as shown for species V. This orientation increases the water ^1^H to local magnetic moment contact distance, reducing the interaction strength, irrespective of the driving dynamics (Néel or Brown) and hence the *r*_1_ suppression is frequency-independent. It is interesting that a single, OS-only, contact interaction is assumed in SPM theory.

In summary, the acidity-induced *r*_1_ suppression arises from a static reduction in the distance of closest approach for the ^1^H in H_2_O, and there is no evidence for limiting proton exchange (even at the lowest pH studied) or other dynamic contribution. Similar arguments are also applicable for H_2_O interaction with bound citrate, in which case increasing ^1^H content will again favor oxygen orientation toward the surface. These findings are contrary to published studies on paramagnetic particles, and their relevance to MR image interpretation and quantitation is discussed in the Conclusions section.

### Dissolution of MNP@APTES in Suspension Monitored by FFC-NMR

The sensitivity of FFC-NMR profiles to the strength and dynamics of the moments also provides a means to monitor the magnetic properties of suspended MNPs during slow dissolution in low pH, lysosome-like, conditions. MNP@APTES suspensions were found to be stable over longer periods at low pH than MNP@cit; hence, they were selected for this model study. Hydrolytic stability may arise from the protonated pendant amines reducing surface accessibility for H^+^. This is consistent with the relaxation mechanism arising from H_2_O, as opposed to H^+^ (or H_3_O^+^), surface interaction. The surface grafted layer may also protect the iron oxide core from dissolution, as has been suggested for silanes following uptake into lysosomes.^39^

**Figure 5:**
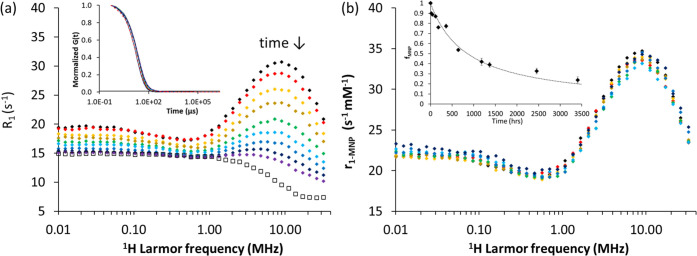
(a) FFC-NMR profiles MNP@APTES, at Fe concentration 0.87 mM, pH 1.8 (initial IS 0.016 M) and 25 ± 3 °C recorded over 4.6 months; 0, 42, 212, 354, 644, 957, 1486, 2222, and 3380 h, and in open markers, *R*_1-d_, the fully dissolved solution. Inset: normalized DLS correlograms of suspensions recorded at the profile measurement times, under the same conditions. (b) MNP contribution to the suspensions relaxivity (r_1-MNP_) over the same time period, extracted directly from the profiles as described in the text. Inset: DLS backscatter count rate (*f*_MNP_) over the dissolution time.

The dissolution of an MNP@APTES suspension of total iron concentration, Fe_tot_, 0.87 mM from atomic absorption spectroscopy, AAS, was evaluated at pH 1.8 and 25 ± 3 °C over a period of 4.6 months using FFC-NMR and DLS. The suspension was adjusted to this pH using 0.1 M HCl, as at this value, the process is slow. The data are shown in Figure 5. The profile recorded on the first day is similar to those shown above for MNP@cit suspensions at similar pH. Over time, the profiles, shown in Figure 5a as *R*_1_ (so not scaled for concentration), are observed to change shape. There is a gradual loss of relaxation across the frequency range and suppression of the high-frequency (superparamagnetic) maximum. Later in the process, the profiles increasingly resemble that of the fully dissolved sample (Figure 5a, open markers). This control data was recorded for a solution of Fe(III) salts, which was prepared by extended dissolution of MNP@APTES with conc. HCl. Full MNP dissolution was confirmed in this case by the absence of any DLS backscatter. The pH of the control was adjusted to 1.8 prior to measurement, and its *R*_1_ values were scaled (following AAS analysis) to the equivalent for a Fe concentration of 0.87 mM, for comparison with MNP@APTES.

DLS measurements for MNP@APTES suspensions at pH 1.8 showed almost no change in hydrodynamic size over the entire period; the correlograms are superimposable, Figure 5b, confirming the absence of any aggregation during dissolution. The backscatter count rate decreased gradually from 419 ± 3 to 100 ± 13 kcps over 4.6 months, Figure 5b, inset. As both *d*_hyd_ and *PDI* are unchanged, the count rate is a measure of the MNP concentration, which gradually falls. Hence, the relative change in the backscattered count rate can be equated to the fraction, *f*_MNP_, of the total Fe remaining in particulate form at a given time. The observed dependence of *f*_MNP_ on time is approximately reciprocal. The suspensions ^1^H relaxation rate, *R*_1-obs_ (=1/*T*_1-obs_), can be expressed as eq 1, assuming independent contributions from MNP and dissolved Fe(III) fractions only.
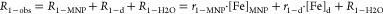
1where *R*_1*-*H_2_0_ is the relaxation rate of water (constant at 0.4 s^–1^); *r*_1-MNP_ is the relaxivity of the MNP@APTES fraction of concentration [Fe]_MNP_; and *r*_1-d_*is* the relaxivity of the dissolved Fe solution of concentration [Fe]_d_. Given that [Fe]_MNP_ = *f*_MNP_*[Fe]_tot_ and so [Fe]_d_ = (1-*f*_MNP_)*[Fe]_tot_, and that [Fe]_Tot_ is 0.87 mM; then, using the *f*_MNP_ values (from DLS) determined as a function of dissolution time, the relaxivity, *r*_1MNP_, of the remaining MNPs can be calculated over that time.

The *r*_1MNP_ values over time, plotted in Figure 5, are almost superimposable. It should be emphasized that to generate this result, the raw *R*_1_ values were only corrected for the dissolved fraction (no scaling). The dispersive features (frequency-dependent inflections) are unchanged, and there is no systematic trend, with only a *ca.* 4–5% variation observed over the full time course. This demonstrates that the relaxivity of the MNP fraction does not change significantly during dissolution; *i.e., M*_S_, *d*, *τ*_N_, and Δ*E*_anis_, as well as the IS *r*_1_ contribution are largely unchanged. However, the concentration of particles gradually decreases. Hence, the NMR data demonstrate that individual MNPs remain magnetically and crystallographically intact until the silanized surface is breached, after which the dissolution is relatively rapid. Interestingly, a significant fraction, *ca.* 20%, of intact unchanged MNP@APTES persist at pH 1.8, even after 4.6 months.

Lévy, Gazeau, and co-workers reported dissolution of dextran-, citrate-, and phosphate-coated MNPs over a slightly less acidic pH range of 2.4–4.7 in 20 mM citrate.^40^ The process was monitored by AAS, DLS, and TEM using interval sampling and by in situ ferromagnetic resonance. At the lowest pH, dissolution was complete after 200 h for citrate, and after 400–600 h for phosphate-coated MNPs. This is more rapid than for MNP@APTES due primarily to the chelating citrate. Detailed kinetic analysis was not undertaken as multiple stages were apparent, and it was also noted that the remaining particles are magnetically intact throughout. In a later study, the same group evaluated poly(acrylic) acid-coated multicore magnetic iron oxide nanoflowers^41^ in “lysosome-mimetic” conditions (pH 4.7, 20 mM citrate), noting in this case loss of magnetic responses by 500 h with rapid initial degradation of the multicore structuring. Interestingly, the inclusion of a gold layer improved long-term chemical stability. In our conditions, combined DLS/FFC-NMR analysis for MNP@APTES suspensions revealed a single clean process extending over a longer time (not complete after 1368 h) at a lower pH. Hence, the silanization and surface charge of the quaternary ammonium groups provide protection against dissolution in highly acidic environments.

As the *f*_MNP_ values provide a measure of the particle concentration as a function of time, a kinetic analysis can be undertaken for MNP@APTES dissolution at pH 1.8 and 25 °C, see Table S1 and Figure S4. The quality of the data is not sufficient for a definitive assignment; however, interestingly, the excellent agreement at a long time, in particular, shows that the change is more consistent with a process that is second order (rather than first) in MNP concentration. First-order dissolution kinetics were described for MNPs coated in citrate (presumably dispersed particles) and oleic acid (presumably dispersed aggregates) in lysosome-mimetic conditions by Gonzalez et al. using Fe determination by AAS following centrifugation, although in that case, the long-time behavior did not adhere well to the model.^42^ The mechanisms are not proven, and indeed they may be dependent on the surface chemistry and environment. However, for MNP@APTES, the data are more dense, the dissolution was slower (600–800 h was reported by Gonzalez), and no transitions were observed between different kinetic regimes at early times. This is good evidence, the first of its kind, that for MNP@APTES suspensions, collisions between particles (which as time progresses have increasingly compromised silane layers) are rate-determining for dissolution.

## Conclusions

It has been shown previously^30^ that isolating the effect of surface interactions on relaxivity for superparamagnetic MNP suspensions requires full particle dispersion. Under conditions where they are fully dispersed and hydrolytically stable, the relaxivity of MNP@cit, MNP@APTES, and MNP@PEG suspensions was shown to be pH-dependent due to acidity-induced suppression of an inner-sphere H_2_O exchange contribution. For MNPs dispersed with hydrophilic ligands, a single average pH-dependent inner-sphere MNP–water contact distance determines *r*_1_ in both the low (Néel) and high (Brownian) frequency ranges. We propose that this arises from the dependence of the average orientation of water molecules on the extent of protonation of the surface, with oxygen orientation toward the surface preferred at a lower pH.

The study suggests options for preparing improved MNP suspensions. For instance, strongly binding bifunctional linkers with silane or catechol-derived headgroups and with pendant carboxylates could provide the requisite acid and chelator stability, and should generate favorable negative ζ. These would provide an amenable alternative to Au coatings^41^ that may both extend the imaging window and moderate detrimental effects of rapid Fe release *in vivo*. Given the presence of relaxivity contributions from a fraction of bare oxide in the case of both silanes used here, APTES and MeO-PEG5000-Si(OMe)_3_, detailed evaluation of the effect of ligand coverage on the pH dependence of *r*_1_ would also be required for such materials.

It is interesting that the pH dependence of *r*_1_ is an order of magnitude stronger than that reported for paramagnetic nanoparticles. This is despite the fact that the exchange involved protons in the paramagnetic case, and so it should be faster. This raises questions about the contributions of the local Fe(III) atomic and the global particle moments to relaxation at different pH, both in the case of superparamagnetic and smaller paramagnetic particles.^10,11,27,28^ The study also highlights the value of FFC-NMR in uncovering key aspects of solvent–particle interactions that determine contrast that are not accessible through fixed-field relaxivity measurements or other techniques. The findings are relevant for developing pH-sensitive imaging agents as the correct relaxivity is essential for quantifying uptake *in vivo* and/or for measuring local pH. They are also relevant in assessing MRI imaging of tumor regions which are often associated with reduced pH, as that may reduce local contrast under *T*_1_-weighting.

During prolonged dissolution at low pH, the profiles of MNP@APTES suspensions showed dramatic changes. However, subtraction of the dissolved Fe contribution demonstrates that the moments of the remaining MNPs are intact throughout dissolution. The findings of this model study are of interest for tracking MNP fate as: (i) the stability of the outer layer may be further improved significantly extending dissolution; (ii) slow dissolution should ensure that free Fe(III) released into the lysosomes reaches homeostasis, so measurement of local MNP concentration or local pH determination may be possible from *R*_1_; (iii) the absence of nanoscale MNP fragments during dissolution should minimize organelle damage; and (iv) the remaining particles retain their relaxivity; hence, as suggested by Gazeau on the basis of *M*_*s*_ retention,^40^ it is likely that the other magnetic responses (AC-field hyperthermia and magnetophoretic capture) also persist over months.

## Methods

### Materials

All chemicals were used as supplied unless otherwise stated:

#### Metal Salts

Iron(III) chloride hexahydrate (Sigma-Aldrich, >99%), iron(III) chloride (Sigma-Aldrich, >99.9%) iron(III) acetylacetonate (Fe(acac)_3_, Sigma-Aldrich, >99.9%, magnesium sulfate (Sigma-Aldrich, 99%), sodium chloride (NaCl, Sigma-Aldrich, ≥99.0%,), sodium hydroxide (NaOH, ≥97.0%, pellets).

#### Ligands

(3-Aminopropyl)triethoxysilane (APTES, Sigma-Aldrich, 99%), sodium citrate tribasic dihydrate(citrate, Sigma-Aldrich, 99%), oleic acid (Sigma-Aldrich, analytical std.), MeO-PEG-Si(OMe)_3_ (PEG5000, Iris-Biotech).

#### Solvents

Methanol (MeOH, Sigma-Aldrich, 99%), ethanol (EtOH, Fisher Scientific, 99%), chloroform (CHCl_3_, Fisher Scientific, 98%), benzyl alcohol (BA, Sigma, >99%), heptane (Sigma, >99%), acetone (Sigma-Aldrich, 99%), tetrahydrofuran (THF, Sigma-Aldrich, 99%). Deionized water was obtained from a Millipore Milli-Q Gradient system fitted with a 0.22 μm Millipak express 20 filter with a resistivity of <18.2 MΩcm.

#### Solutions

Iron standard (Sigma-Aldrich, 1001 ± 4 mg mL^–1^ in water), hydrochloric acid (HCl, Sigma-Aldrich, 37% in water)

### Particle Synthesis and Stabilization

#### MNP@BA

Magnetic nanoparticle suspensions in benzyl alcohol (MNP@BA)were prepared using the following modification to the Pinna method.^31^ In brief, Fe(acac)_3_ (1.00 g, 2.83 mol) was dissolved in benzyl alcohol (20 mL) in a 100 mL three-neck round-bottom flask. The solution was purged with N_2_ for 10 min before being refluxed under N_2_ at 205 °C for 7 h. During the synthesis, the color of the solution/suspension changes from red to dark red and to black at the concentration used. Full details are provided in our previous work.^43^

#### MNP@OA Suspensions

MNP@OA suspensions in heptane were prepared for size analysis by TEM using homogenized MNP@BA in benzyl alcohol (5 mL).

#### MNP@APTES Suspensions

Homogenized MNP@BA in benzyl alcohol (5 mL) was crashed out using a magnet and then washed three times with acetone. The pellet was then resuspended in APTES (125 μL, 0.53 mM), chloroform (1.5 mL), and water (29 μL, 1.6 mmol) and was shaken for 3 h. EtOH was added, and the particles were crashed out with a magnet. The supernatant was removed, and the particles were washed with acetone before being resuspended in water (1.5 mL) and a trace of HCl (1–5 μL of 1 M is sufficient to generate a stable final suspension) and shaken for 1 h. Finally, the suspensions were centrifuged three times (10 min, 16.2 RCF) with the supernatant removed after each centrifugation, before final resuspension in water with light agitation. This serves to increase the monodispersity of the MNPs suspension by removing larger aggregates and larger particles if present; the resulting suspensions have excellent long-term colloidal stability (by DLS).

#### MNP@Cit Suspensions

Homogenized MNP@BA in benzyl alcohol (4 mL) was crashed out using a magnet, then washed three times with acetone, and the pellet was then resuspended in a sodium citrate in water solution (5.2 mM, 20 mL). The suspensions were then centrifuged three times (10 min, 16.2 RCF) with the supernatant removed after each centrifugation, before final resuspension in water with light agitation. The yield of the APTES stabilization step is 30–50% for different batches. This stabilization involves hydrolysis and condensation steps, and in our hands, it is more susceptible to the starting conditions, especially the age of the APTES reagent. So the lower yield than for MNP@cit is not surprising.

#### MNP@PEG Suspensions

Homogenized MNP@BA in benzyl alcohol (1 mL) was crashed out using a magnet and then washed three times with acetone. The pellet was then resuspended in water (2 mL), NaOH (100 μL, 0.1 mM), and MeO-PEG-Si(OMe)_3_ (MW 5 kDa, 282 μL, 70 mg mL^–1^ in THF). The suspension was heated in a water bath at 70 °C for 1 h. Water (2.9 mL) was added followed by CHCl_3_ (5 mL). The suspension was shaken to transfer the MNP@PEG into the CHCl_3_ layer, and the water layer was removed. The CHCl_3_ layer was washed four times with water, with the water layer being removed after each washing. The CHCl_3_ was allowed to evaporate, then the dried MNP@PEG pellet was resuspended in water (5 mL), and the suspension was then centrifuged two times (10 min, 16.2 RCF) with the supernatant removed after each centrifugation, before final resuspension in water with light agitation.

### Transmission Electron Microscopy

Transmission electron microscopy (TEM) imaging was performed using the FEI Tecnai G2 20 TWIN 200 keV transmission electron microscope in the Conway Institute in UCD. MNP@OA (Fe 2 mM, 7 μL) were spotted on to a TEM grid (Formvar and carbon films on a 400 μm copper grid, Agar scientific). Size analysis for MNP@OA was performed manually using ImageJ software. For MNP@OA, the mean size was determined by measuring the longest axis of each particle and the axis orthogonal, the average of these two values was taken as the diameter of the particle, and the aspect ratio was calculated by dividing the orthogonal axis by the longest axis. The raw data were fitted to a log-normal distribution using OriginPro 9 software to provide the *d*_TEM_ value and uncertainty quoted.

### Fast-Field-Cycling Nuclear Magnetic Resonance

The ^1^H spin-lattice relaxation times, *T*_1_, of MNP suspensions were measured as a function of Larmor frequency, ν_L_, using a Spinmaster FFC-2000 Fast Field Cycling NMR Relaxometer (Stelar, Mede, Italy). Typically, relaxation profiles were recorded from 0.01 to 40 MHz, *T*_1_ was calculated from four scans, and each point was measured twice and a final mean value was taken. Temperature control was provided by a Spinmaster Variable Temperature Controller (Stelar, Mede, Italy), and all measurements were made at 22 ± 0.1 °C. For ν_L_ > 12 MHz, a nonpolarized (NP/S) sequence was used, and for ν_L_ < 12 MHz, a pre-polarized (PP/S) sequence was used. The acquisition field was 16.1 MHz for all measurements.

The spin-lattice relaxivity (*r*_1_, expressed in mM^–1^ s^–1^) is calculated as follows
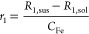
where *R*_1,sus_ and *R*_1,sol_ are the spin-lattice relaxation rates (*R*_1_, units, s^–1^) of the MNP suspension and pure solvent, respectively, and *C*_Fe_ is Fe concentration of Fe in the suspension in mM. We have estimated the error in the Fe determination as *ca*. 2%.^44^ This determines the uncertainty in the *r*_1_ values, as the error in the *R*_1_ values is significantly smaller, at <1%.

Dissolution of MNP@APTES was conducted over 4.6 months at pH 1.8, with time points at 0, 42, 212, 354, 644, 957, 1486, 2222, and 3380 h, corresponding to 0.00, 0.06, 0.29, 0.48, 0.88, 1.31, 2.04, 3.04, and 4.63 months.

### Dynamic Light Scattering and Zeta Potential

Dynamic light scattering (DLS) measurements were performed at 25 °C using a Malvern NanoZS (Malvern Instruments, Malvern U.K.). Typically, the iron concentration of each sample was between 0.25 and 2 mM. Analysis is done using Dispersion Technology software (v. 4.10, Malvern Instruments; Worcestershire, U.K.) using the multiple narrow modes algorithm based upon a non-negative least-squares fit. For all suspensions presented, the *PDI* values were low and the cumulants fit matched the data very well; hence, the *Z*-ave value is taken as a measure of *d*_hyd_ throughout. Zeta potential (ζ) measurements were performed at 25 °C on a Malvern NanoZS (Malvern Instruments, Malvern U.K.) using the M3-PALs approach. All DLS and zeta potential measurements were run in triplicate. In all cases, the three values were in very close agreement, and the average was used.

### Flame Atomic Absorption Spectroscopy

The iron content in samples was determined using flame atomic absorption spectroscopy (AAS). Spectra were recorded on a SpectrAA 55B atomic absorption spectrometer (Varian, U.K.). A Fe-cathode lamp (248.3 nm) was used with a high-temperature air/acetylene flame. The limit of detection of the instrument is 0.05 ppm. Typically, the MNP suspension (between 50 and 100 μL) was placed in a volumetric flask (50–100 mL), to which HCl (1.5 mL, 12 M) and deionized water (1 mL) were added. The samples were left for 2 h before being made up to the mark with nitric acid (1 M). A calibration curve was made using iron standards (0.1–5 mg L^–1^), which were prepared by diluting a 1000 mg L^–1^ Fe standard in nitric acid (1 M).

### pH Measurements

The pH of MNP suspensions was measured using an LE pH Electrode LE438-IP67 (Mettler Toledo) with the Five Easy pH meter (Mettler Toledo). A three-point calibration was used prior to each set of pH measurements. InLab Solutions (Mettler Toledo) calibration solutions with pH 4.01, 7.00, and 9.21 were used for calibration. The pH probe was washed with deionized water between measurements.

## Supporting Information Available

Additional FFC-NMR profiles; additional DLS data; and kinetics analysis of MNP@APTES dissolution (PDF)
