# Supplementary material for: pH Dependence of MRI Contrast in Magnetic Nanoparticle Suspensions Demonstrates Inner-Sphere Relaxivity Contributions and Reveals the Mechanism of Dissolution
Source: Langmuir. 2023 Feb 3;39(6):2171–81. doi: 10.1021/acs.langmuir.2c02621 (PMC9933532; doi:10.1021/acs.langmuir.2c02621)
Supplement: Supplementary file 1 — la2c02621_si_001.pdf [file la2c02621_si_001.pdf]

pH-dependence of MRI-contrast in magnetic nanoparticle suspensions demonstrates inner-sphere relaxivity contributions and reveals the mechanism of dissolution

Eoghan M[ac]Mahon[a] and Dermot F. Brougham[a,*]

[a] School of Chemistry, University College Dublin, Belfield, Dublin 4, Ireland

[*] **Corresponding author**

[*] dermot.brougham@ucd.ie

SUPPLEMENTARY INFORMATION

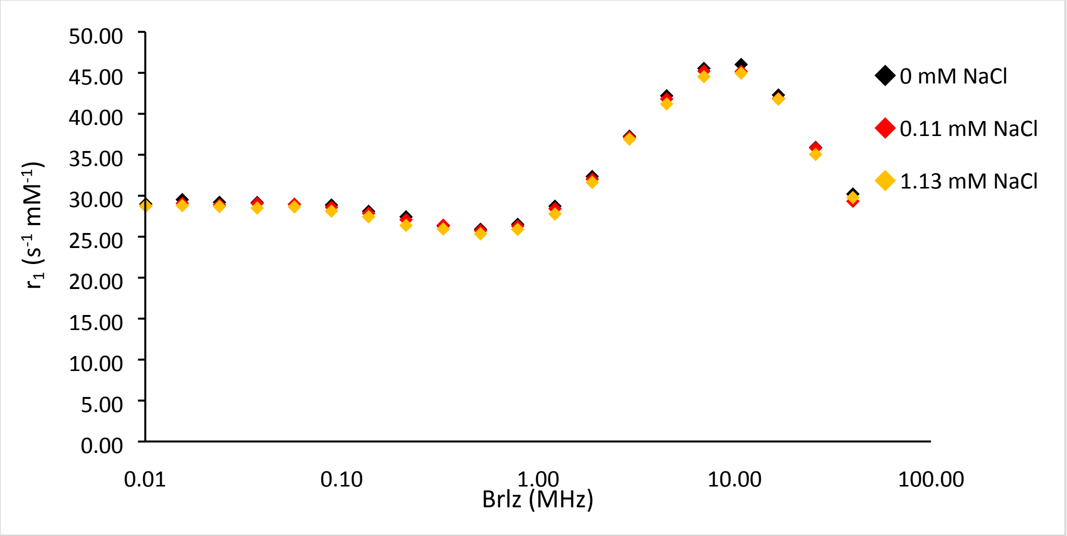

Figure s1. Profiles of MNP@cit at pH 7.1 as a function of NaCl concentration. The fact that the profiles are almost superimposable and the hydrodynamic sizes were unchanged (the pH of all these suspensions was within 0.1 units of pH 7.1 and the d_{hyd} values were within 0.5 nm of 14.5 nm, and all *PDI* values were < 0.19) confirms that the effects shown in Figure 3 are due to pH, and not ionic strength.

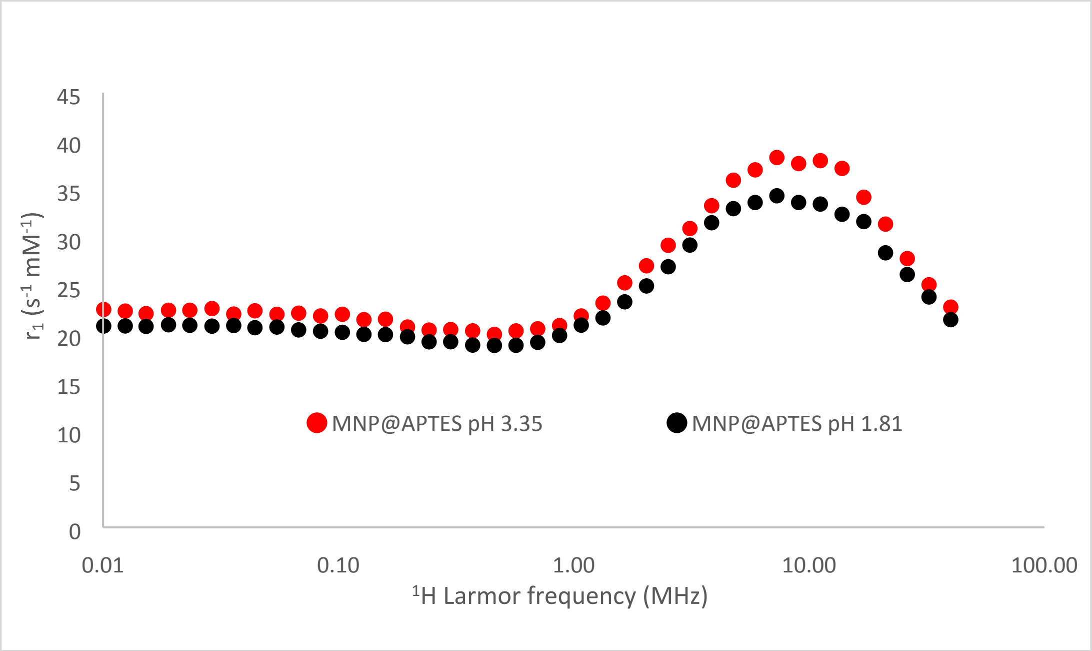

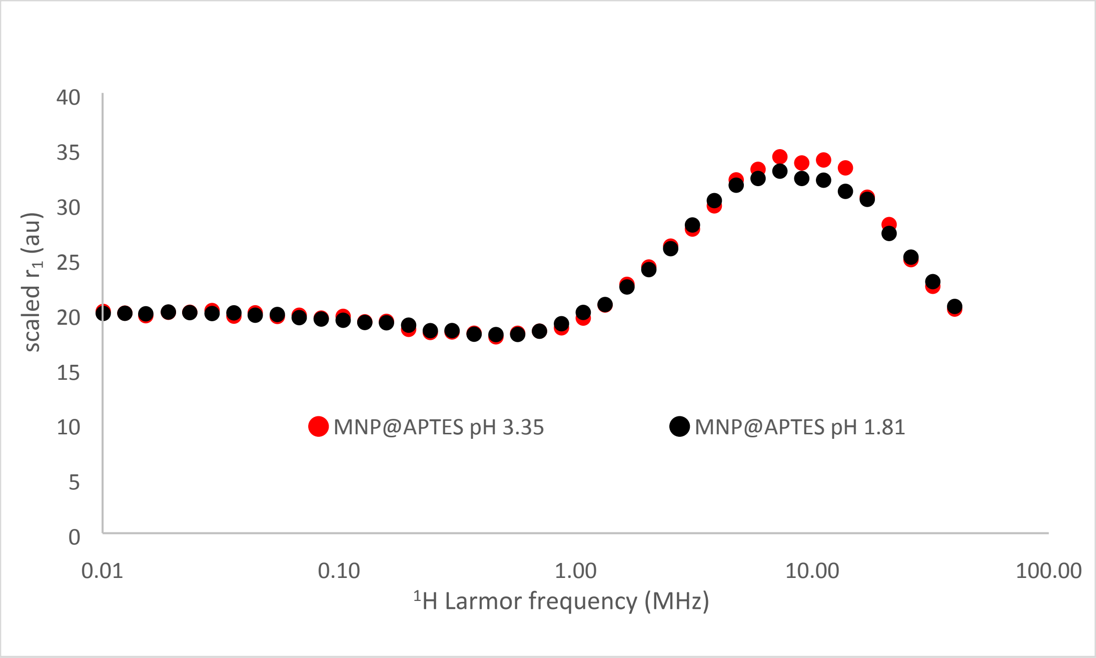

Figure s2. Upper, FFC-NMR profiles of MNP@APTES. Lower, the same profiles scaled to a common r_1 (20 au at 0.01 MHz).

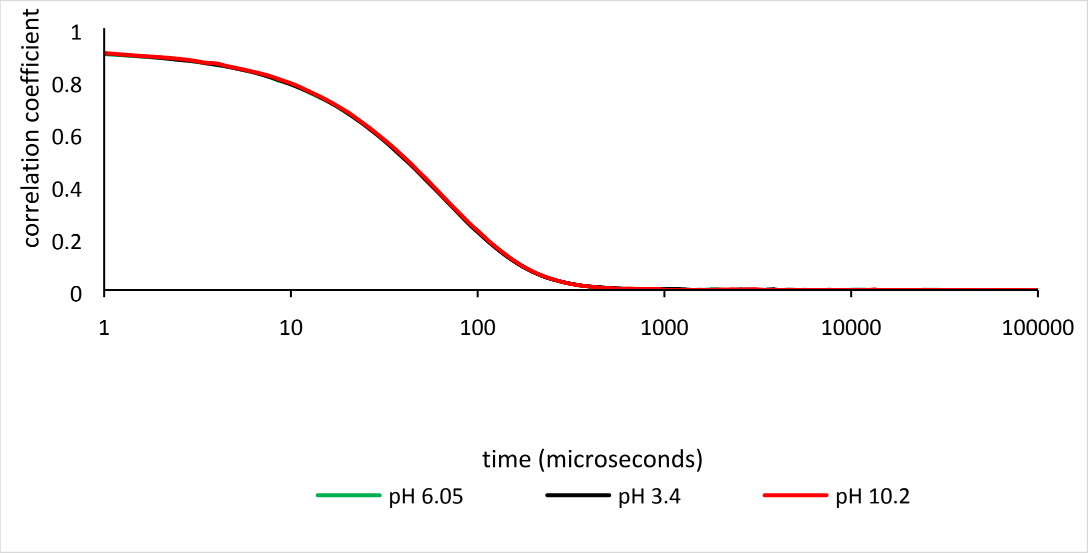

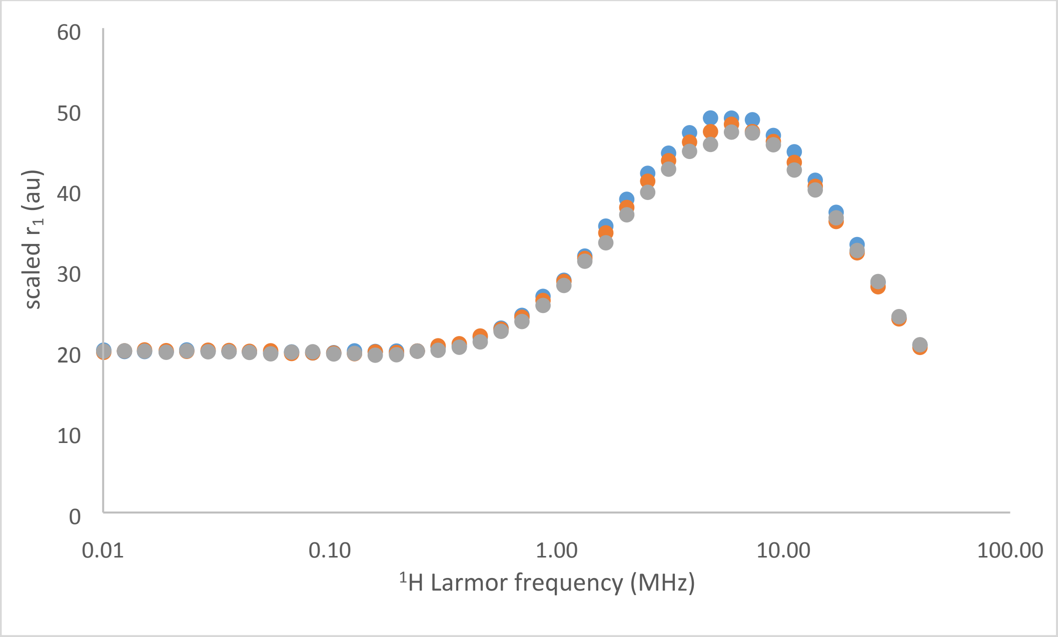

Figure s3. Upper, DLS correlograms for MNP@PEG at pH 3.4, 6.1 and 10.2 demonstrating that d_{hyd} is unchanged over the pH range studied. Lower, the profiles recorded for MNP@PEG at pH 3.4, 6.1 and 10.2 scaled to a common r_1 (20 au at 0.01 MHz)

Table s1. Kinetics analysis of MNP@APTES dissolution.

		Time (hr)	Experimental f_{MNP}	MNPs per mL	2nd order 1/[MNP]	1st order ln[MNP]
		0	1	3.99E+13	2.51E-14	3.13E+01
Total Fe (mol L^{-1})	8.74E-04	20	0.898997	3.59E+13	2.79E-14	3.12E+01
Total Fe (mol mL^{-1})	8.74E-07	41.5	0.88873	3.55E+13	2.82E-14	3.12E+01
TEM radius (m)	4.40E-09	118.5	0.870583	3.47E+13	2.88E-14	3.12E+01
MNP volume (m^3)	3.57E-25	182.5	0.760745	3.04E+13	3.29E-14	3.10E+01
MNP density (kg m^{-3})	4900	360.5	0.773161	3.09E+13	3.24E-14	3.11E+01
		644.5	0.537966	2.15E+13	4.66E-14	3.07E+01
MNP mass (kg)	1.75E-21	1183	0.42001	1.68E+13	5.97E-14	3.05E+01
Maghemite molecular mass (g mol^{-1})	159.69	1368	0.389685	1.56E+13	6.43E-14	3.04E+01
Moles of maghemite per MNP	1.09488E-20	2456	0.326886	1.30E+13	7.66E-14	3.02E+01
Moles of Fe per MNP	2.18975E-20	3405	0.238777	9.53E+12	1.05E-13	2.99E+01
Number of MNP per mL	3.99E+13					

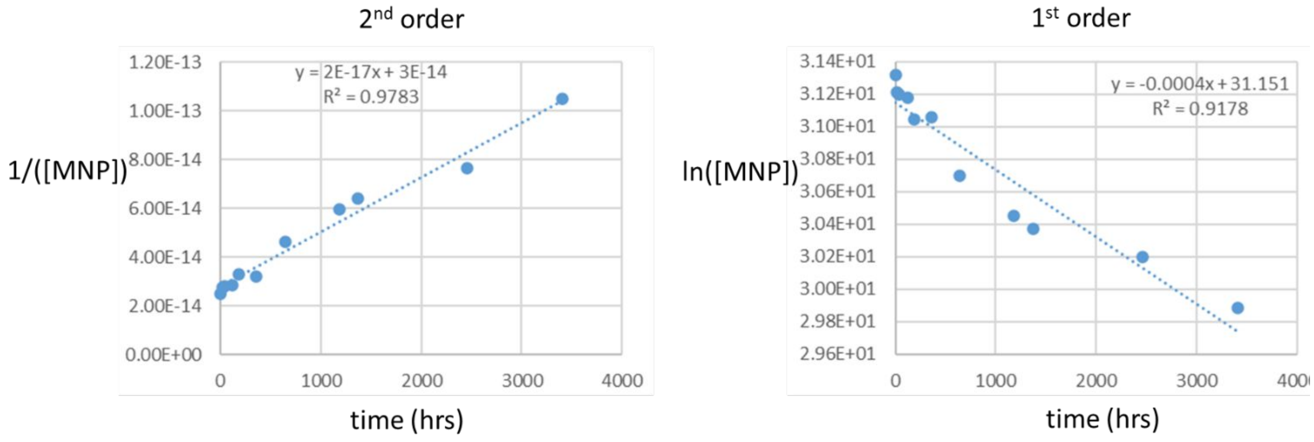

Figure s4. Kinetics analysis of MNP@APTES dissolution.
